# SOCIAL ENGAGEMENT AMONG COMMUNITY-DWELLING OLDER ADULTS: A QUALITY-OF-LIFE ANALYSIS

**DOI:** 10.1093/geroni/igad104.2382

**Published:** 2023-12-21

**Authors:** Alisha Thompson, Scott Wilks, Rhonda Bardales

**Affiliations:** LSU, Baton Rouge, Louisiana, United States; Louisiana State University, Baton Rouge, Louisiana, United States; Louisiana State University, Baton Rouge, Louisiana, United States

## Abstract

Older adults are living longer without sufficient efforts from health and social service systems to improve their quality of life (QoL). QoL is a nuanced, dynamic aspect to analyze. Literature shows that higher levels of social engagement provide a range of benefits to physical, mental, and cognitive health, all broad indicators of overall QoL. Social engagement comprises the person’s subjective experience of the richness of their relationships. The purpose of this study was to examine empirically the connection among this richness of relationships — specifically social engagement and perceived quality of relationships — and overall QoL among older adults. The study used a large sample (N=746) of non-institutionalized adults, ages 65 and older, from the nationally representative, AARP Social Engagement and Brain Health Survey. Two primary independent variables (IVs) were quantifiable measures of social engagement and perceived quality of social relationships; covariance controlled for several demographic factors (e.g., sex, ethnicity, income) and general health measures of physical, mental, and emotional health. The outcome variable was a composite score from a popular QoL measure. Analyses involved bivariate regression for the primary IVs, then multivariate OLS regression with each IV to predict quality of life. Results showed that QoL significantly regressed with predictors of social engagement (positively); each health variable (positively); unmarried status; ethnicity of Black/African American or Asian; and male gender. These results enhance our understanding of the relationship between social engagement, relationship quality, and QoL in older adults, particularly those who identify within the aforesaid demographic brackets. Implications are discussed.

